# Supplementary material for: The General Public and Young Adults’ Knowledge and Perception of Palliative Care: A Systematic Review
Source: Healthcare (Basel). 2024 May 7;12(10):957. doi: 10.3390/healthcare12100957 (PMC11121430; doi:10.3390/healthcare12100957)
Supplement: Supplementary file 1 [file healthcare-12-00957-s001.zip › Table_S1.pdf]

S1: Reviewers‘ key findings

Erstautor	Land	Studie	Key Findings: Reviewer 1	Key Findings: Reviewer 2
Abuelzeet [1]	Jordanien	deskriptives Querschnittsdesign der Bevölkerung<br>PaCKS	• viele haben noch nie was von Palliativmedizin gehört -> große Wissenslücke in der Bevölkerung<br>• eher kein Wissen über Palliativmedizin, wenn sie antworten, dann aber meist richtig<br>• Wissen besser, wenn sie vorab schon von Palliativmedizin gehört haben<br>• Alter macht keinen Unterschied	• Kaum Wissen zur Palliativmedizin<br>• Nur wenige haben überhaupt schon von Palliativmedizin gehört
Akiyama [2]	Japan	Intervention: Öffentlichkeitsarbeit zur Info über Palliativmedizin über 3 Jahre<br>Prä-post Befragung per Fragebogen von Tumorpatient:innen, trauernden Angehörigen und der Bevölkerung -> Querschnittserhebung	• Informationsmaterial und Öffentlichkeitsarbeit können eine Unterstützung das Wissen über Palliativmedizin zu erweitern.<br>• Dies kann Betroffenen Sicherheit geben.	• Öffentlichkeitsarbeit führt zu Wissen zur Palliativmedizin
Al-Atiyyat [3]	international	integratives Review	• Es könnte einen Zusammenhang zwischen Wissen übe Palliativmedizin und deren Anforderung bestehen.<br>• Daher ist Öffentlichkeitsarbeit und Information notwendig. Die Öffentlichkeit, Betroffene und Gatekeeper müssen sensibilisiert werden. Das kann auch in nationale Strategien aufgenommen werden. Und dabei sollte es um alle Aspekte von Palliativmedizin gehen und nicht nur um end-of-life.<br>• Pflegende und Gesundheitsmitarbeiter können stärkend sein.	• Ressource: Mitarbeiter im Gesundheitswesen<br>• Nationale Guidelines müssen Palliativmedizin repräsentieren
Alkhudairi [4]	Saudi-Arabien	Querschnittsstudie der allgemeinen Öffentlichkeit<br>Fragebogen über Google Docs gesendet	• Wissen über und Bewusstsein von Palliativmedizin ist eher gering.<br>• Signifikant ist der Bildungsabschluss, aber nicht das Alter.<br>• Informationen der Öffentlichkeit über Palliativmedizin sind notwendig.	• Bildung korreliert positiv mit Wissen
Bazargan [5]	USA	Querschnittsstudie	• Nicht-hispanische Weißen war Palliativmedizin und HC mehr bekannt<br>• Muss jemand nahes versorgt werden, sind Palliativmedizin und HC bekannter.<br>• Bekanntheitsgrad von Palliativmedizin und HC korreliert mit Alter, Geschlecht, Bildung und Einkommen	• Ethnische Minderheiten sind schlechter informiert<br>• Geschlecht und Alter sind Determinanten für Wissen
Benini [6]	Italien	Zufallsstichprobe der Bevölkerung	• Die meisten haben von Palliativmedizin schon gehört, aber nur ein knappes Viertel meint eine genauere Vorstellung darüber zu haben<br>• Am häufigsten: Linderung von Schmerzen und Steigerung der Lebensqualität<br>• Es bedarf mehr Infos über Palliativmedizin	• Wahrnehmung: Schmerzen reduzieren, Lebensqualität steigern<br>• Aber: Kein nuanciertes Wissen

S1: Reviewers‘ key findings

Erstautor	Land	Studie	Key Findings: Reviewer 1	Key Findings: Reviewer 2
Bergman [7]	Niederlande	Querschnittsstudie	• Die meisten haben schon mal von PV gehört, aber nur knapp die Hälfte meinen zu wissen, was es ist.<br>• Wissen, dass es nicht nur Menschen mit onkologischen Erkrankungen betrifft und dass es nicht ausschließlich um die letzten Lebenswochen geht, ist vorhanden, allerdings eher gering<br>• Wissen muss erweitert werden	• Fehlendes nuancierter Wissen
Bichu [8]	Indien	Querschnittsstudie mit Bewohnern der Region	• Viele TN haben von Palliativmedizin gehört und haben Wissen darüber; viele haben Palliativmedizin aber auch schon durch ein Familienmitglied was Palliativmedizin benötige/ bekommen hat, kennengelernt -> Repräsentativität?	• Familienmitglieder sind wichtige Ressourcen für Wissen
Bin Ahmed [9]	Saudi-Arabien	Prävalenzstudie	• Die meisten haben noch nichts von Palliativmedizin gehört und die was gehört haben, können es aber nur zu einem geringen Anteil einordnen	• Fehlendes nuanciertes Wissen
Boucher [10]	USA	Interviews und Fokusgruppen, Inhaltsanalyse; Zufallsstichprobe	• Wissenslücken über Palliativmedizin (Finanzierung, Initiierung)<br>• Wahrnehmung, dass so etwas im Gesundheitssystem nicht umsetzbar wäre<br>• Die Pat. Die Erfahrung damit haben, berichten, dass ein Zeitdruck bleibt und das Personal nicht mehr Zeit für die Versorgung hätte.<br>• Weitere Information und Edukation ist notwendig.	• Barriere: Finanzierung, Integration der Palliativmedizin in den klinischen Alltag
Chandra [11]	Indien	Prä/post Interventionsstudie	• Bewusstsein für Palliativmedizin hat nach der Intervention deutlich zugenommen -> von 0 auf > 62%; aber das Interesse hat abgenommen. (Es gab auch welche bei denen es nicht zugenommen hat, wahrscheinlich waren sie nicht bei allen Interventionsangeboten dabei, was die Aussage über den Effekt verstärkt)<br>• Aufklärung wichtig und effektiv, aber man muss dranbleiben, einmalige Kampagnen reichen nicht aus.	• Aufklärung und Wissensvermittlung erhöht Bewusstsein für Palliativmedizin<br>• Interventionen zielgerichtet
Collins [12]	Australien	Querschnittsstudie mit Interviews Phänomenologischer Rahmen	• Palliativmedizin wird mit verminderter Versorgung im Sinne von verminderter Kuration/ Therapie/ Hoffnung in Verbindung gebracht -> negative Assoziation<br>• Frühzeitige Integration bedarf guter Info und Aufklärung, auch in der Gesellschaft	• Angst vor Verlust von Hoffnung und Fürsorge<br>• Angst, keine bedarfsgerechte Therapie zu erhalten
Collins [13]	Australien	Querschnittsbefragung; gemeindebasierte Stichprobe, freiwillige Meldung auf Anzeige	• Fehlendes/ falsches Wissen über Palliativmedizin führt dazu, dass potentielle Betroffenen es nicht nutzen.<br>• Daher bedarf aus Information/ Aufklärung der Gesellschaft.	• Informationen führen zu erhöhter Inanspruchnahme<br>• Öffentlichkeitsarbeit

S1: Reviewers‘ key findings

Erstautor	Land	Studie	Key Findings: Reviewer 1	Key Findings: Reviewer 2
Dionne-Odom [14]	USA	Nutzung populationsbasierter Daten eines vorherigen nationalen Surveys; pflegende Angehörige wurden befragt	• Deutliche Wissenslücken über Palliativmedizin<br>• Ethnische Herkunft und Bildungsabschluss waren relevant (Minderheiten, Menschen ohne Hochschulabschluss wussten weniger)<br>• Palliativmedizin und HC wird gleichgesetzt<br>• Palliativmedizin = Tod<br>• Aufklärung und Information notwendig	• Palliativmedizin = Hospiz<br>• Palliativmedizin = Tod
Dixe [15]	Portugal	Querschnittsstudie; online Befragung; Gesundheitspersonal und Nicht-Gesundheitspersonal	• Es gab signifikante Unterschiede bei den beiden Gruppen.<br>• Lebensqualität (GP) vs. Chronische und fortschreitende Erkrankungen (NGP)<br>• Allgemein bedarf es weiteres Wissen über Information	• Health Care professionals wissen mehr<br>• Bevölkerung weiß zu wenig zu Palliativmedizin
Dosani [16]	Kanada	Palliativmedizin Patient:innen einer örtlichen Klinik<br>Fragebogen und Interviews	• Teilergebnis: Lücke im Wissen über Palliativmedizin -> aber wichtig für eine gute Versorgung, daher weitere Information notwendig	• Fehlendes nuanciertes Wissen
Dyal [17]	USA	Querschnitterhebung; drei Stichproben (gemeindenah, Florida, USA)	• Altersunterschiede bei Wissen; junge Erwachsene wissen weniger um Einbezug Angehörige und um Symptomreduzierung.<br>• Ländliche TN sehen Palliativmedizin eher als Aufgeben<br>• Info und Aufklärung wichtig	• Junge Erwachsene wissen weniger um die Relevanz der Angehörigen in der Versorgung<br>• Angst vor Aufgeben
Fliedner [18]	Schweiz	Qualitativer Ansatz; Besucher einer Palliativmedizin Ausstellung an 6 Standorten; Kartenbefragung mit zwei offenen Fragen	• Palliativmedizin wurde überwiegend positiv assoziiert<br>• Interprofessionalität<br>• Palliativmedizin eher mit Lebensende assoziiert<br>• Wissen um Verständnis kann bei Aufklärung und Umgang helfen	• Palliativmedizin = Versorgung am Lebensende<br>• Wissen muss dissmeminiert werden
Gill [19]	International	Systematisches Review mit interpretativer Analyse	• Unterschiede im Wissen über Palliativmedizin; gering v.a. bei Männern, jungen Menschen und ethnischen Minderheiten<br>• Palliativmedizin verbunden mit Aufgeben, Tod, aber auch mit natürlichem Tod<br>• Persönliche Erfahrungen verbessern das Verständnis<br>• Es bedarf weiterer Information	• Negative Assoziationen<br>• Junge Erwachsene wissen weniger als ältere Menschen
Gopal [20]	Indien	Fragebogenstudie; Allgemeinbevölkerung und Gesundheitspersonal (bestimmtes Krankenhaus)	• Allgemeinbevölkerung weniger Wissen<br>• Es braucht Info und Aufklärung	• Wissensdefizite<br>• Öffentlichkeitswirksame Kampagnen gefordert
Grant [21]	Literatur genutzt die US-Population beschreiben	Scoping Review	• Palliativmedizin weniger bekannt als HC, allerdings auch da falsche Annahmen<br>• Palliativmedizin häufig mit Lebensende verbunden<br>• Aufklärung notwendig	• Palliativmedizin = Lebensende

S1: Reviewers' key findings

Erstautor	Land	Studie	Key Findings: Reviewer 1	Key Findings: Reviewer 2
He [22]	China	Mixed Methods; Fragebogenentwicklung (Wissenschaftler und Praktiker); Befragte: Bevölkerung	• Weniger Verfügbarkeit -> weniger Wissen<br>• Nachfrage auf dem Land höher<br>• Weitere Rolle spielt: Kultur, Tabuisierung von Tod, Konflikte Arzt/ Patient<br>• Kultursensibel Ansätze zur Förderung von Palliativmedizin notwendig	• Strukturen vorhanden = Wissen zur Palliativmedizin gesteigert<br>• Kampagnen sollten kulturelle Determinanten der Zielpopulation inkludieren
Heckel [23]	Deutschland	Online Survey; Bürger	• Palliativmedizin ist bekannt, wird mit umfassender Versorgung plus Angehörige in Verbindung gebracht.<br>• Es überwiegt aber vor allem die Annahme, dass es für Sterbende ist.	• Assoziation: Lebensende
Hidalgo-Andrade [24]	Ecuador	Querschnittsstudie; Schneeballsystem über Social Media; online Fragebogen	• Wissenslücke wird aufgezeigt<br>• Geschlecht, Bildung, Beruf und Erfahrung als Caregiver stehen in Bezug zum Wissen über Palliativmedizin	• Soziale Determinanten bestimmen Wissen
Hong [25]	USA	Analyse der Suchfragen bei Google Trends	• mehr Suchinteresse an HC als an Palliativmedizin<br>• Korrelation zwischen Palliativmedizin Suchanfragen und Palliativmedizin Programmen<br>• Keine Korrelation mit HC Suchanfragen	• In Ländern, in denen Palliativmedizin flächendeckend verankert ist, gibt es mehr Wissen
Hsu [26]	Taiwan	Querschnittstudie; Fragebogen; Ältere Menschen die eine Gesundheitsuntersuchung erhalten haben.	• Viele haben von Hospice Care gehört, aber nur wenige von Gesundheitspersonal<br>• Hospice Care wird nicht mit DNR gleichgesetzt<br>• Höhere Bildung korreliert mit mehr Wissen über Hospice Care	• Health Care Professionals informieren kaum zur Palliativmedizin
Huang [27]	Taiwan	Querschnittsdesign mit sozioökologischem Modell	• Wissen über Palliativmedizin relevant für Gespräche über Versorgungswünsche am Lebensende	• Aktive Auseinandersetzung mit End-of-Life Orders und Wissen zur Palliativmedizin
Huemer [28]	Deutschland, Österreich, Schweiz	Auswertung der Suchanfragen über Google Trends zu Richtlinien, Gesetzen und Verordnungen	• Interesse an Palliativmedizin stieg mit Verfügbarkeit und Umsetzung von Gesetzen und Maßnahmen zur Förderung<br>• Sicherung Patientenautonomie kann Suchaktivitäten zu assistiertem Suizid verringern<br>• Palliativmedizin sinnvoll um Bewusstsein für Versorgung am Lebensende zu schärfen	• Nationale Integration von Strukturen der Palliativmedizin führt zu mehr Wissen
Hugar [29]	Florida	Patienten mit einem Blasentumor einer bestimmten Klinik; Fragebogen und Interview	• TN meinen ein hohes Wissen über Palliativmedizin zu haben, es zeigte sich aber, dass es häufig Fehlannahmen sind. So wird Palliativmedizin mi HC und Tod assoziiert, sehen es in erster Linie als emotionale und psychische Unterstützung oder als „Aufgeben"<br>• Dennoch würden sie es selbst für sich oder Angehörige wählen<br>• Es bedarf mehr Info und Aufklärung	• Fehlendes nuanciertes Wissen<br>• Falsche Selbsteinschätzung

S1: Reviewers‘ key findings

Erstautor	Land	Studie	Key Findings: Reviewer 1	Key Findings: Reviewer 2
Huo [30]	USA	Nutzung eines landesweiten Surveys	• Viele TN wussten nichts über Palliativmedizin, der Anteil war bei den Hispanics noch höher<br>• Älterer Bevölkerung hat mehr Wissen über Palliativmedizin als < 50-jährige<br>• Zudem gab es Missverständnisse über Palliativmedizin<br>• Es bedarf weiterer Aufklärung	• Ethnische Minderheiten wissen weniger über Palliativmedizin<br>• Ältere Menschen wissen mehr als jüngere
Igarashi [31]	Japan	Querschnittsfragebogen	• Wahrnehmung von Opioiden und Palliativmedizin<br>• viele Fehlwahrnehmungen<br>• besseres Palliativmedizin Wissen führte zu besserer Wahrnehmung, das wiederum gab mehr Sicherheit in Bezug auf Versorgung bei Tumorerkrankungen<br>• Aufklärung wichtig	• Assoziationen zur Palliativmedizin negativ und falsch
Kahveci [32]	Türkei	Querschnittstudie; Angehörige von palliativen Patienten; Fragebogen kurz nach Aufnahme auf die Palliativstation	• Die meisten hatten keine Vorkenntnisse<br>• Palliativmedizin wird als lebensverlängernd und kurativ angesehen<br>• Infos meist erst, wenn Angehörige damit in Kontakt kommen oder im Freundeskreis	• Wissen wird durch persönlichen Kontakt zu palliativmedizinischen Strukturen erhöht
Kozlov [33]	USA	TN über Amazons Mechanical Turk (Plattform für Verhaltens- und Kognitionsforschung); PaCKS und demographischer Fragebogen	• Viel „ich weiß nicht“ Antworten<br>• fehlendes Wissen über Palliativmedizin<br>• Frauen und Ältere besseres Wissen	• Geschlecht und Alter korrelieren positiv mit Wissen
Lei [34]	China	Interviews mit Senioren aus Pflegeheimen	• Wissen über Palliativmedizin war gering<br>• bei Aufklärung und Vorhandensein würden sie es nutzen<br>• Kinder übernehmen Verantwortung, daher sollten sie aufgeklärt werden<br>• Wichtig: Palliativmedizin ist nicht Euthanasie (wurde verwechselt)	• Terminologie unbekannt<br>• Fehlendes Wissen
Lu [35]	USA	Querschnittstudie mit Daten aus einem routinemäßigen nationalen Survey	• Bewusstsein für und Einstellungen zu Palliativmedizin beeinflussen das Nutzen von Palliativmedizin<br>• Viele haben noch nichts von Palliativmedizin gehört<br>• Bildung, Geschlecht und Einkommen korrelieren mit dem Wissen über Ziele von Palliativmedizin<br>• Es bedarf weiterer Aufklärung	• Soziale Determinanten<br>• Inanspruchnahme-Verhalten wird durch Wahrnehmung getriggert
MacLeod [36]	Neuseeland	Bevölkerungsrepräsentative Stichprobe; über ein Marktforschungsunternehmen; online Umfrage	• HC und Palliativmedizin waren bekannt und es gab ein Bewusstsein dafür<br>• Alter und Geschlecht haben einen Einfluss; jüngere Menschen und Männer haben eher weniger Wissen über zugängliche Hospizversorgung in der Region;<br>• Alter korreliert mit Wissen über Möglichkeiten von Palliativmedizin<br>• Es bedarf weitere Information und Aufklärung über das Thema	• Jüngere Menschen wissen weniger<br>• Männliches Geschlecht korreliert negativ mit Wissen

S1: Reviewers‘ key findings

Erstautor	Land	Studie	Key Findings: Reviewer 1	Key Findings: Reviewer 2
Maddalena [37]	Kanada	qualitative Arbeit; Fokusgruppen; gezielte Stichprobe von 6 pflegenden Angehörigen von Sterbenden/ Verstorbenen aus einer „black community“	• begrenztes Wissen über Palliativmedizin Versorgung<br>• familienzentrierte Pflege als Grund für das fehlende Hinzuziehen von Palliativmedizin<br>• Informationen dringen nicht bis zu ihnen vor<br>• Es bedarf auch kultureller Anpassungen.	• Kulturelle Determinanten beeinflussen Inanspruchnahme-Verhalten
Mahmudur [38]	Bangladesch	Querschnitt	• Junge Menschen haben nur begrenztes Wissen<br>• Kein klares Konzept	• Junge Menschen haben nur wenig Wissen zur Palliativmedizin<br>• Nur wenige erfahren von Gesundheitspersonal über Palliativmedizin
Mallon [39]	UK	Querschnittstudie; PaCKS; junge Erwachsene (18-29 Jahre), die an einer Universität in UK studieren	• Viele TN hatten Wissen über Palliativmedizin bzw. schon mal davon gehört<br>• Männer haben weniger Wissen<br>• Wissen darüber, dass es nicht ausschließlich für Tumorerkrankte und Ältere ist<br>• bei Zeitpunkt und weiterem eher unsicher	• Männliches Geschlecht negativ korreliert<br>• Zeitgerechte Integration wird nicht wahrgenommen
Mallon [40]	UK	qualitative Arbeit; Interviews; Zufallsstichprobe junger Erwachsener (18-29 Jahre) -> vorheriger TN am online Fragebogen (Mallon_2), Bereitschaft an Interview teilzunehmen, wer mit Ort und Zeit einverstanden war	• Palliativmedizin als Comfort Care gesehen, mit Bezug zu fehlenden Heilungschancen und Tod<br>• Negative Assoziationen: Hoffnungslosigkeit<br>• TN sagen selbst, es braucht mehr und vor allem klare Information und Wissen<br>• junge Menschen sollten in Gesundheitsthemen einbezogen werden	• Inklusion junger Menschen<br>• Dissemination von Wissen muss gesteigert werden
Manu [41]	USA	Deskriptive Querschnittsstudie mit älteren Erwachsenen, die von einer bestimmten Klinik ambulant betreut werden.	• HC vertrauter als Palliativmedizin<br>• Pflege eines sterbenden Angehörigen ging nicht mit einer positiveren Einstellung gegenüber Palliativmedizin und HC oder mehr Vertrautheit mit dem Thema einher<br>• Es bedarf mehr Information der älteren Menschen	• Persönliche Erfahrungen beeinflussen Wahrnehmung
MacIlfatrick [42]	Nordirland	Querschnittserhebung, Fragebogen (mit offenen Fragen) postalisch und online; Mitglieder des Patient and Client Councils (Organisation für Patienten und Klienten, die sich bei der Entwicklung von Sozial- und Gesundheitsfragen beteiligen möchte und für Studien bereitstehen)	• Begrenztes Wissen über Palliativmedizin<br>• Wissen und Einstellungen korreliert mit Geschlecht (Frauen mehr und positiver) und vorausgegangen Erfahrungen mit Palliativmedizin<br>• Haupthindernisse für Bewusstseinsbildung: Angst, mangelnde Erfahrung und Wahrnehmung, dass Ressourcen fehlen	• Fehlendes nuanciertes Wissen<br>• Barrieren: Angst, Hoffnungslosigkeit, mangelnde Ressourcen
McIlfatrick [43]	Nordirland	qualitative Arbeit	• Grundsätzliches Wissen über Palliativmedizin vorhanden, meist aus eigenen Erfahrungen raus<br>• Verbunden mit Tod/ Sterben und Versorgung in den letzten Lebenstagen<br>• Erfahrung schafft Verständnis; das sollte aber grundsätzlich durch Info, Aufklärung, Programme gesteigert werden	• Assoziation zum Lebensende

S1: Reviewers‘ key findings

Erstautor	Land	Studie	Key Findings: Reviewer 1	Key Findings: Reviewer 2
MacIlfatrick [44]	Nordirland	Mixed Methods, Querschnittstudie und Interviews (s.o.)	• Geschlecht (Frauen), höher Bildung, verheiratet, Alter (Älter) korrelieren mit mehr Bewusstsein für Palliativmedizin<br>• Missverständnisse: Palliativmedizin für Menschen, die höchsten noch 6 Monate zu leben haben<br>• Es sollte mehr über Palliativmedizin gesprochen werden, aber es wird ein gesellschaftliches Tabu wahrgenommen	• Soziale Determinanten<br>• Assoziation zum Lebensende
Merkuri [45]	Albanien	Fragebogen; repräsentatives Sample	• viele haben nichts von Palliativmedizin gehört und kennen keine lokalen Palliativmedizin Versorger<br>• Fehlannahmen über Erkrankungen, v.a. AIDS wird als Indikation angegeben<br>• Es bedarf deutlich mehr Info und Aufklärung	• Palliativmedizin eher unbekannt<br>• Kein Wissen, wie man Strukturen in Anspruch nehmen kann
Miyashita [46]	Japan	TN aus Stadtdistrikten; einstündige öffentliche Aufklärungsvorträge in allen Distrikten; TN sollten vorher und nachher Fragebögen ausfüllen	• Nach den Veranstaltungen konnten sich mehr Personen ein häusliches Versterben vorstellen.<br>• Verbesserung bei: Einstellung zur Pflege am Lebensende incl. Symptommanagement<br>• Veränderungen, v.a. bei Männern gesehen<br>• Infoveranstaltung kann Einfluss auf Möglichkeit der häuslichen Pflege haben	•
Noh [47]	USA	pflegende Angehörige von chronisch oder schwer kranken älteren Menschen wurden persönlich und individuell mit einer Broschüre über Palliativmedizin informiert; Prä-Post-Test; PaCKS	• prä-post signifikant unterschiedlich<br>• prä: signifikanter Unterschied zwischen weißen und schwarzen TN, post kein Unterschied mehr<br>• gezielte Information kann Wissen über Palliativmedizin verbessern	•
O’Connor [48]	Australien	Deskriptive Querschnittserhebung; Personen einer Gemeinde	• gutes Wissen über Palliativmedizin<br>• Einstellungen, Haltungen, Emotionen eher positiv<br>• Weniger Wissen über Sterbehilfe, Lebensverlängerung -verkürzung, Palliativmedizin nur in Krankenhäusern	• Ressourcen für Palliativmedizin weniger bekannt
Patel [49]	International	Scoping Review	• Allgemein eher geringes Wissen über Palliativmedizin<br>• mehr Wissen bei Frauen, über 40-jährigen, Erfahrung im privaten Umfeld, Mitarbeiter im Gesundheitssystem<br>• wenig Wissen, es bestehen Fehlannahmen -> Info und Aufklärung wichtig	• Soziale Determinanten beeinflussen das Wissen
Ramos-Vera [50]	Südamerika	Querschnittsstudie; Daten aus einer nationalen Erhebung; derselbe Survey wie bei Lu, ob gleiches Jahr nicht klar (hier 2018, bei Lu keine Angaben)	• Selbstfürsorge Selbstwirksamkeit stehen in Zusammenhang mit Wissen über und Haltung zu Palliativmedizin<br>• Kann wichtig für Pläne zur Verbesserung von Zugang und Adhärenz sein	• Persönliche Haltung beeinflusst das Wissen und die Wahrnehmung

S1: Reviewers' key findings

Erstautor	Land	Studie	Key Findings: Reviewer 1	Key Findings: Reviewer 2
Sanjo [51]	Japan	Querschnittstudie; Allgemeinbevölkerung und Hinterbliebene von 12 Palliativstationen; Fragebogen	• Hinterbliebene haben ein besseres Wissen und Vorstellungen bezüglich Palliativmedizin<br>• Beide Gruppen sehen Palliativstationen als Ort bei dem aufs Sterben gewartet wird.<br>• Trotz Erfahrung fehlt Wissen, allgemeine Info und Aufklärung nötig	• Persönliche Erfahrungen korrelieren mit dem Wissen zur Palliativmedizin<br>• Fehlendes nuanciertes Wissen
Schweighart [52]	Deutschland	qualitatives Design	• Viele TN gaben Wissen über Palliativmedizin an, es flossen viele Vorstellungen in die Ergebnisse ein.<br>• Nur wenige gaben eine frühzeitige Integration an, Verbindung meist mit Versterben; Symptomlinderung als zentrales Ziel, aber auch LQ als Ziel benannt; Interprofessionalität war weniger bekannt, sowie ambulante Versorgungsmöglichkeiten<br>• Palliativmedizin wird überwiegend positiv gesehen	• Zeitgerechte Integration wird kaum wahrgenommen<br>• Assoziation vor allem zum Lebensende
Shah [53]	Pakistan	Querschnittstudie; Pflegekräfte; stationäre und ambulante Versorgung eines bestimmten Krankenhauses und häusliche Palliativversorgung	• Mehrheit findet, dass Tumorpatienten über Diagnose aufgeklärt werden sollten.<br>• Wissen über Palliativmedizin korreliert mit Bildung, Alter (hier haben eher junge Pflegende mehr Wissen -> in Ausbildung neu?) und Erfahrung<br>• Palliativmedizin sollte allen Menschen angeboten werden<br>• Ca. die Hälfte wusste es, damit es mehr wissen bedarf es weiterer Infoprogammen.	• Soziale Determinanten<br>• Aufklärung, dass Palliativmedizin für alle Patientengruppen verfügbar sein sollte
Shalev [54]	USA	Querschnittstudie; Daten einer nationalen Erhebung im Staat New York, erhoben von einer Marketinggruppe	• Wissen über Palliativmedizin und HC vorhanden, allerdings lücken- und fehlerhaft<br>• Hauptkomponenten werden nicht genannt.<br>• Es bedarf mehr Info und Aufklärung	• Fehlendes nuanciertes Wissen
Shen [55]	USA	Befragung der Allgemeinbevölkerung; bei z.B. Gesundheitsmessen, Kirchen, Neujahrsfeiern, in Gemeindezentren; PaCKS und KOCO	• eher geringer Bekanntheitsgrad von Palliativmedizin<br>• Alter war eher negativ und Bildung positiv mit Bewusstsein für Palliativmedizin assoziiert<br>• Es bedarf kulturgerechter Infoprogramme.	• Bildung beeinflusst Wissen zur Palliativmedizin<br>• Anpassen von Kampagnen an kulturelle Determinanten
Smith [56]	USA	Rekrutierung der TN über E-Mail-Listen, Gemeindekalender und soziale Medien; 60-minütige virtuelle Infoveranstaltung über Palliativmedizin; Fragebogen prä/post; PaCKS + weitere Fragen zur Annahme von Palliativmedizin	• Post Werte stiegen deutlich<br>• Virtuelle Infoveranstaltung hat positiven Effekt	• Informationsveranstaltungen erhöhen Wissen, auch niederschwellig online
Taber [57]	USA	Querschnittserhebung; Daten aus nationalem Survey (wieder HINT wie bei Lu und Ramos-Vera; 2018 wir bei Ramos-Vera)	• Auch TN die angegeben haben viel über Palliativmedizin zu wissen, hatten Wissenslücken, ungenaue Angaben oder negative Assoziationen<br>• Aufklärung und Info notwendig	• Diskrepanz Selbsteinschätzung und tatsächliches Wissen
Trivedi [58]	USA	wieder aus dem HINT 2018	• Viele geben kein Wissen über Palliativmedizin an bzw. noch nie davon gehört zu haben.<br>• Ältere Menschen, höherer Bildungsstand, Frauen und Weiße wissen mehr	• Soziale Determinanten beeinflussen das Wissen

S1: Reviewers‘ key findings

Erstautor	Land	Studie	Key Findings: Reviewer 1	Key Findings: Reviewer 2
Westerlund [59]	Schweden	online Umfrage; Bevölkerungsstichprobe; randomisierte Stichprobe aus der schwedischen Datenerhebungsstelle; Info über Studie per Mail	• Meist keine oder wenige Infos über Palliativmedizin<br>• Frauen, Ältere, höhere Bildung, Arbeit im Gesundheitswesen oder Erfahrung im soziale Umfeld-> mehr Wissen über Palliativmedizin<br>• Haupthindernisse für Info über Palliativmedizin: Angst, Scham und Tabu sowie fehlende Relevanz, aber auch Mangel an Infos<br>• Vorschläge zur Information auf verschiedenen Wegen (z.B. Medien wie Fernsehen oder schon in der Schule) wurden gemacht	• Soziale Determinanten beeinflussen Wissen<br>• Barrieren: Angst, Tabuisierung
Zimmermann [60]	Kanada	Daten aus Umfrage in der kanadischen Öffentlichkeit	• Teilweise viel Wissen über Palliativmedizin<br>• Bei hohem richtigen Wissen -> weniger Ängste<br>• Niedriges Wissen kann zum Aufrechterhalten von falschen Annahmen über Palliativmedizin führen<br>➔ Info und Aufklärung notwendig	• Wissen determiniert Wahrnehmung

S1: Reviewers' key findings

1. *Abuelzeet AY, Zeilani R, Othman EH.* Public awareness and knowledge of palliative care in Jordan. Int J Palliat Nurs. 2023:29(6):264–73.
2. *Akiyama M, Hirai K, Takebayashi T, Morita T, Miyashita M, Takeuchi A, et al*. The effects of community-wide dissemination of information on perceptions of palliative care, knowledge about opioids, and sense of security among cancer patients, their families, and the general public. Supportive Care in Cancer. 2016;24(1):347–56.
3. *al-atiyyat, Nijmeh & Gazo, Mohammed. (2015).* Public Awareness toward Palliative Care: Integrative Literature Review. Journal of Health, Medicine and Nursing. 19. 67-70.
4. *Alkhudairi, HasanMohammed. (2018).* General public awareness, knowledge, and beliefs toward palliative care in a Saudi population. Journal of Nature and Science of Medicine. 2. 10.4103/JNSM.JNSM_37_18.
5. *Bazargan M, Cobb S, Assari S, Kibe LW.* Awareness of Palliative Care, Hospice Care, and Advance Directives in a Racially and Ethnically Diverse Sample of California Adults. AMERICAN JOURNAL OF HOSPICE & PALLIATIVE MEDICINE. 2021;38(6):601–9.
6. *Benini F, Fabris M, Pace DS, Vernò V, Negro V, De Conno F, et al*. Awareness, understanding and attitudes of Italians regarding palliative care. Ann Ist Super Sanita. 2011;47(3):253–9.
7. *Bergman TD, van der Plas AGM, Pasman HRW, Onwuteaka-Philipsen BD*. Awareness and Actual Knowledge of Palliative Care Among Older People: A Dutch National Survey. J Pain Symptom Manage. 2023 Sep;66(3):193-202.e2.
8. *Bichu PB, Abyrai K, Shaliet R.* Awareness and Self Reported Utilization Pattern of Community Palliative Care Units in a Rural Area in Central Kerala. CONTEMPORARY SOCIAL SCIENCES. 2020;29(2):17–26.
9. *Bin Ahmed IA, Bin Ahmed AA, Bin Ahmed IA, AlFouzan SK.* A descriptive online survey about the knowledge of palliative care residents of Saudi Arabia has compared to the general worldwide population. Saudi Med J. 2020;41(5):537-541.
10. *Boucher NA, Bull JH, Cross SH, Kirby C, Davis JK, Taylor DH*. Patient, Caregiver, and Taxpayer Knowledge of Palliative Care and Views on a Model of Community-Based Palliative Care. J Pain Symptom Manage. 2018;56(6):951–6.
11. *Chandra A, Sarkar S, Adinarayanan S, Balajee KL.* Impact of Health Awareness Campaign in Improving the Perception of the Community about Palliative Care: A Pre- and Post-intervention Study in Rural Tamil Nadu. Indian J Palliat Care. 2016;22(4):467-476.
12. *Collins A, McLachlan SA, Philip J*. Community knowledge of and attitudes to palliative care: A descriptive study. Palliat Med. 2020;34(2):245–52.

13. *Collins A, McLachlan SA, Philip J.* Initial perceptions of palliative care: An exploratory qualitative study of patients with advanced cancer and their family caregivers. Palliat Med. 2017;31(9):825–32.
14. *Dionne-Odom JN, Ornstein KA, Kent EE*. What do family caregivers know about palliative care? Results from a national survey. Palliat Support Care. 2019;17(6):643–9.
15. *Dixe M dos A, Santo ID de O, Lopes S, Catarino H, Duarte S, Querido A, et al*. Knowledge and myths about palliative care among the general public and health care professionals in Portugal. Int J Environ Res Public Health. 2020;17(13):1–12.
16. *Dosani N, Bhargava R, Arya A, Pang C, Tut P, Sharma A, et al*. Perceptions of palliative care in a South Asian community: Findings from an observational study. BMC Palliat Care. 2020;19(1).
17. *Dyal BW, Yoon SL, Powell-Roach KL, Li DM, Kittelson S, Weaver M, Krieger JL, Wilkie DJ.* Perceptions of Palliative Care: Demographics and Health Status Among the General Population in Florida and the United States. Am J Hosp Palliat Care. 2023;28:10499091231186819.
18. *Fliedner MC, Zambrano SC, Eychmueller S.* Public perception of palliative care: a survey of the general population. Palliat Care Soc Pract. 2021;15:26323524211017546.
19. *Gill A*. A systematic review and critical interpretive synthesis of public perceptions of palliative care. 2020 [zitiert 17. Juli 2023]; Verfügbar unter: https://hull-repository.worktribe.com/OutputFile/4223153
20. *Gopal KS, Archana PS.* Awareness, Knowledge and Attitude about Palliative Care, in General, Population and Health Care Professionals in Tertiary Care Hospital. Int J Sci Stud 2016;3(10):31-35.
21. *Grant MS, Back AL, Dettmar NS.* Public Perceptions of Advance Care Planning, Palliative Care, and Hospice: A Scoping Review. J Palliat Med. 2021;24(1):46–52.
22. *He L, Liao H, Pan Y, Lu Y, Xu M, Liu M, et al.* Current public perception of, and barriers to, palliative care in China: a mixed-methods study. thelancet.com [Internet]. 2022 [zitiert 17. Juli 2023]; Verfügbar unter: https://www.thelancet.com/journals/lanonc/article/PIIS1470-2045(22)00407-7/fulltext
23. *Heckel M, Schweighardt S, Baron M, Peters J, Ostgathe C*. Die Wahrnehmung der Palliativmedizin in der Öffentlichkeit - erste Ergebnisse eines Online-Survey [268]. 13 Kongress der Deutschen Gesellschaft für Palliativmedizin. August 2020;21.
24. *Hidalgo-Andrade P, Mascialino G, Miño D, Mendoza M, Marcillo AB*. Knowledge of palliative care in Ecuador. Int J Environ Res Public Health. 2021;18(9).

25. *Hong YR, Huo JH.* Trends in US Population Interest in Palliative Care and Its Association With Prevalence of Palliative Care Programs in US States. J Pain Symptom Manage. 2020;60(1):E89–91.
26. *Hsu CP, Chen HW, Lee SY, Tsou MT*. Knowledge and Attitude toward Hospice Palliative Care among Community-Dwelling Aged Taiwanese-Analysis of Related Factors. Int J Gerontol. 2012;6(2):105–11.
27. *Huang YL, Yates P, Thorberg FA, Wu CJ.* Influence of social interactions, professional supports and fear of death on adults' preferences for life-sustaining treatments and palliative care. Int J Nurs Pract. 2022;28(4).
28. *Huemer M, Jahn-Kuch D, Hofmann G, Andritsch E, Farkas C, Schaupp W, et al.* Trends and Patterns in the Public Awareness of Palliative Care, Euthanasia, and End-of-Life Decisions in 3 Central European Countries Using Big Data Analysis From Google: Retrospective Analysis. J Med Internet Res. 2021;23(9).
29. *Hugar LA, Geiss C, Chavez MN, Gore LR, Thirlwell S, Reblin M, et al.* Exploring knowledge, perspectives, and misperceptions of palliative care: A mixed methods analysis. Urologic Oncology: Seminars and Original Investigations. 2023;41(7):327.e19-327.e26.
30. *Huo J, Hong YR, Grewal R, Yadav S, Heller IW, Bian J, et al.* Knowledge of Palliative Care Among American Adults: 2018 Health Information National Trends Survey. J Pain Symptom Manage. 2019;58(1):39-47.e3.
31. *Igarashi A, Miyashita M, Morita T, Akizuki N, Akiyama M, Shirahige Y, et al.* A Population-Based Survey on Perceptions of Opioid Treatment and Palliative Care Units: OPTIM Study. American Journal of Hospice and Palliative Medicine. 2014;31(2):155–60.
32. *Kahveci K, Gokcinar D.* Knowledge About Palliative Care in the Families of Patients. Acta Medica Mediterranea. 2014;30(6):1369–73.
33. *Kozlov E, McDarby M, Reid MC, Carpenter BD.* Knowledge of Palliative Care Among Community-Dwelling Adults. American Journal of Hospice and Palliative Medicine. 2018;35(4):647–51.
34. *Lei L, Lu Y, Gan Q, Hu Z, Luo Y*. Awareness and Perceptions of Palliative Care Among the Elderly: A Qualitative Study. J Palliat Care. 2022;37(2):204–12.
35. *Lu X, Liu J.* Factors Influencing Public Awareness of and Attitudes Toward Palliative Care: A Cross-Sectional Analysis of the 2018 HINTS Data. Front Public Health. 2022;10.
36. *MacLeod RD, Thompson R, Fisher JW, Mayo K, Newman N, Wilson DM.* New Zealanders' knowledge of palliative care and hospice services. New Zealand Medical Journal. 2012;125(1348):51–60.

37. *Maddalena V, Bernard WT, Davis-Murdoch S, Smith D.* Awareness of Palliative Care and End-of-Life Options Among African Canadians in Nova Scotia. Journal of Transcultural Nursing. 2013;24(2):144–52.
38. *Mahmudur Rahman AHM, Khan HA*. Fundamental perceptions about palliative care among young generations living in Dhaka city, Bangladesh: A short survey. Prog Palliat Care. 4. Mai 2017;25(3):121–5.
39. *Mallon A, Hasson F, Casson K, Slater P, McIlfatrick S.* Young adults understanding and readiness to engage with palliative care: extending the reach of palliative care through a public health approach: a qualitative study. BMC Palliat Care. 2021;20(1).
40. *Mallon A, Slater P, Hasson F, Casson K, McIlfatrick S.* What do young adults know about palliative care? A cross-sectional survey. Public Health. 2021;191:78–84.
41. *Manu E, Mack-Biggs TL, Vitale CA, Galecki A, Moore T, Montagnini M.* Perceptions and Attitudes About Hospice and Palliative Care Among Community-Dwelling Older Adults. American Journal of Hospice and Palliative Medicine. 2013;30(2):153–61.
42. *McIlfatrick S, Hasson F, McLaughlin D, Johnston G, Roulston A, Rutherford L, et al.* Public awareness and attitudes toward palliative care in Northern Ireland. BMC. 2013:12(1):34.
43. *McIlfatrick S, Noble H, McCorry NK, Roulston A, Hasson F, McLaughlin D, Johnston G, Rutherford L, Payne C, Kernohan G, Kelly S, Craig A.* Exploring public awareness and perceptions of palliative care: a qualitative study. Palliat Med. 2014 Mar;28(3):273-80.
44. *McIlfatrick S, Slater P, Beck E, Bamidele O, McCloskey S, Carr K, et al.* Examining public knowledge, attitudes and perceptions towards palliative care: a mixed method sequential study. BMC Palliat Care. 2021;20(1).
45. *Merkuri L, Dervishi D*. Knowledge, attitudes and behaviors of the community about palliative care. Medicus, 2022:6(1), 11–18.
46. *Miyashita M, Sato K, Morita T, Susuki M.* Effect of a population-based educational intervention focusing on end-of-life home care, life-prolonging treatment and knowledge about palliative care. Palliat Med. Juni 2008;22(4):376–82.
47. *Noh H, Lee LH, Won C.* Educational intervention to improve palliative care knowledge among informal caregivers of cognitively impaired older adults. Palliat Support Care. 2021;19(5):515–23.
48. *O'Connor M, Breen LJ, Watts KJ, James H, Goodridge R.* A Tripartite Model of Community Attitudes to Palliative Care. American Journal of Hospice and Palliative Medicine. 2019;36(10):877–84.

49. *Patel P, Lyons L.* Examining the Knowledge, Awareness, and Perceptions of Palliative Care in the General Public Over Time: A Scoping Literature Review. American Journal of Hospice and Palliative Medicine. 2020;37(6):481–7.
50. *Ramos-Vera C, Krikorian A, Vallejos Saldarriaga J.* Factors related to positive attitudes toward palliative care: Direct and indirect effects of self-care self-efficacy, knowledge, and beliefs. Palliat Support Care. 2022;27:1-7.
51. *Sanjo M, Miyashita M, Morita T, Hirai K, Kawa M, Ashiya T, et al.* Perceptions of Specialized Inpatient Palliative Care: A Population-Based Survey in Japan. J Pain Symptom Manage. 2008;35(3):275–82.
52. *Schweighart SM.* Die Wahrnehmung der Palliativmedizin in der Öffentlichkeit [Internet]. opus4.kobv.de. 2022 [zitiert 17. Juli 2023]. Verfügbar unter: https://opus4.kobv.de/opus4-fau/frontdoor/deliver/index/docId/20741/file/DissertationSusannaMariaSchweighart.pdf
53. *Shah S, Qaisar F, Azam I, Mansoor K*. Perceptions, knowledge and attitudes towards the concept and approach of palliative care amongst caregivers: a cross-sectional survey in Karachi, Pakistan. BMC Palliat Care. 2020;19(1).
54. *Shalev A, Phongtankuel V, Kozlov E, Shen MJ, Adelman RD, Reid MC*. Awareness and Misperceptions of Hospice and Palliative Care: A Population-Based Survey Study. American Journal of Hospice and Palliative Medicine. 2018;35(3):431–9.
55. *Shen JJ, Dingley C, Yoo JW, Rathi S, Kim SK, Kang HT, et al.* Sociocultural factors associated with awareness of palliative care and advanced care planning among Asian populations. Ethn Dis. 2020;30(3):459–68.
56. *Smith G, Bragg A, Kling S.* Improving Public Knowledge and Attitudes About Palliative Care Through Virtual, Community-Based Education (Sch439). JPSM. 2023:65(5), e600.
57. *Taber JM, Ellis EM, Reblin M, Ellington L, Ferrer RA*. Knowledge of and beliefs about palliative care in a nationally-representative U.S. Sample. PLoS One. 2019;14(8).
58. *Trivedi N, Peterson EB, Ellis EM, Ferrer RA, Kent EE, Chou WYS.* Awareness of Palliative Care among a Nationally Representative Sample of U.S. Adults. J Palliat Med. 2019;22(12):1578–82.
59. *Westerlund C, Tishelman C, Benkel I, Fürst CJ, Molander U, Rasmussen BH, et al.* Public awareness of palliative care in Sweden. Scand J Public Health. 2018;46(4):478–87.
60. *Zimmermann C, Wong JL, Swami N, Pope A, Cheng Y, Mathews J, Howell D, Sullivan R, Rodin G, Hannon B, Moineddin R, Le LW*. Public knowledge and attitudes concerning palliative care. BMJ Support Palliat Care. 2021 Oct 7:bmjspcare-2021-003340.
