# Supplementary material for: The General Public and Young Adults’ Knowledge and Perception of Palliative Care: A Systematic Review
Source: Healthcare (Basel). 2024 May 7;12(10):957. doi: 10.3390/healthcare12100957 (PMC11121430; doi:10.3390/healthcare12100957)
Supplement: Supplementary file 1 [file healthcare-12-00957-s001.zip › Table_S2.pdf]

Table S2: Search strings

Database	Search string	Last searched
Medline via PubMed	(population [tiab] OR "general public" [tiab] OR community [tiab] OR people [tiab] OR "young people" [tiab] OR society [tiab] OR adolescent* [tiab] OR "young adult*" [tiab])<br>AND<br>(campaign* OR information OR education* OR research OR implementation OR "education intervention" OR "public health" OR "health awareness campaign*" OR engagement OR program* OR participation* OR "public health practice" OR "health promotion" OR "information dissemination")<br>AND<br>palliat* [ti]<br>AND<br>(perception* [tiab] OR belief* [tiab] OR awareness [tiab] OR knowledge [tiab] OR attitude* [tiab] OR stigma* [tiab] OR stereotyp* [tiab] OR myth* [tiab] OR "health knowledge, attitudes, practice" OR "public opinion" OR "education, medical, continuing" OR "community health services")<br>NOT<br>(physician* OR nurse* OR pharmacist* OR resident* OR staff OR "general practitioner*" OR "health care provider*" OR "medical staff, hospital" OR “physician assistant*”).	17 July 2023

Web of Science	population OR "general public" OR community OR people "young people" OR society OR adolescent* OR "young adult*" (All Fields) and campaign* OR information OR education* OR research OR implementation OR "education intervention" OR "public health" OR "health awareness campaign*" OR engagement OR program* OR participation* OR "public health practice" OR "health promotion" OR "information dissemination" (All Fields) and palliat* (Title) and perception* OR belief* OR awareness OR knowledge OR attitude* OR stigma* OR stereotyp* OR myth* OR "public opinion" OR "community health services" (All Fields) not physician* OR nurse* OR pharmacist* OR resident* OR staff OR "general practitioner*" OR "health care provider*" OR “physician assistant*” (All Fields).	17 July 2023
Google Scholar	English:<br>allintitle: palliative (perception OR perceptions OR knowledge OR attitude OR attitudes OR belief OR beliefs OR awareness OR stigma OR stigmata) population OR young OR people OR public OR adult OR adolescent OR adolescents OR community OR campaign<br><br>German:<br>allintitle: Palliativmedizin Empfindung OR Wahrnehmung OR Wissen OR Haltung OR Bewusstsein OR Stigma OR Stigmatisierung OR Kenntnisse OR Meinung	17 July 2023
